# Intrauterine Growth Restriction Alters Mouse Intestinal Architecture during Development

**DOI:** 10.1371/journal.pone.0146542

**Published:** 2016-01-08

**Authors:** Camille M. Fung, Jessica R. White, Ashley S. Brown, Huiyu Gong, Jörn-Hendrik Weitkamp, Mark R. Frey, Steven J. McElroy

**Affiliations:** 1 Division of Neonatology, Pediatrics, University of Utah, Salt Lake City, Utah, United States of America; 2 Division of Neonatology, Pediatrics, University of Iowa, Iowa City, Iowa, United States of America; 3 Division of Neonatology, Pediatrics, Vanderbilt University, Nashville, Tennessee, United States of America; 4 Department of Pediatrics and Department of Biochemistry and Molecular Biology, University of Southern California Keck School of Medicine and The Saban Research Institute at Children's Hospital Los Angeles, Los Angeles, California, United States of America; Cincinnati Children's Hospital Medical Center, UNITED STATES

## Abstract

Infants with intrauterine growth restriction (IUGR) are at increased risk for neonatal and lifelong morbidities affecting multiple organ systems including the intestinal tract. The underlying mechanisms for the risk to the intestine remain poorly understood. In this study, we tested the hypothesis that IUGR affects the development of goblet and Paneth cell lineages, thus compromising the innate immunity and barrier functions of the epithelium. Using a mouse model of maternal thromboxane A_2_-analog infusion to elicit maternal hypertension and resultant IUGR, we tested whether IUGR alters ileal maturation and specifically disrupts mucus-producing goblet and antimicrobial-secreting Paneth cell development. We measured body weights, ileal weights and ileal lengths from birth to postnatal day (P) 56. We also determined the abundance of goblet and Paneth cells and their mRNA products, localization of cellular tight junctions, cell proliferation, and apoptosis to interrogate cellular homeostasis. Comparison of the murine findings with human IUGR ileum allowed us to verify observed changes in the mouse were relevant to clinical IUGR. At P14 IUGR mice had decreased ileal lengths, fewer goblet and Paneth cells, reductions in Paneth cell specific mRNAs, and decreased cell proliferation. These findings positively correlated with severity of IUGR. Furthermore, the decrease in murine Paneth cells was also seen in human IUGR ileum. IUGR disrupts the normal trajectory of ileal development, particularly affecting the composition and secretory products of the epithelial surface of the intestine. We speculate that this abnormal intestinal development may constitute an inherent “first hit”, rendering IUGR intestine susceptible to further injury, infection, or inflammation.

## Introduction

Gestation has long been recognized as a critical period for programming the eventual adult phenotype. Thus it is a time when adverse *in utero* conditions can induce alterations in the original blueprint. One of the most basic outcomes that can be assessed during gestation is the size of an infant relative to its gestational age. Infants with intrauterine growth restriction (IUGR) are traditionally defined as weighing less than the 10^th^ percentile for his or her gestational age. Etiologies of this growth defect may include primary deficits in placental function such as when pregnancy-induced hypertension interferes with placental blood flow. They may also include secondary placental dysfunction resulting from adverse maternal behaviors such as cigarette smoking or environmental influences including maternal under-nutrition and stress. Regardless of the etiology, infants born with IUGR are at significantly increased risk of short- and long-term morbidities affecting multiple organ systems, including the cardiovascular, endocrine, pulmonary, and neurologic systems[1]. This increased risk of morbidity also affects the gastrointestinal tract[1].

Infants with IUGR often display poor intestinal function. They commonly exhibit feeding intolerance[2, 3], and they are at an increased risk for neonatal intestinal diseases such as necrotizing enterocolitis (NEC) compared to their normally grown counterparts[4–6]. Bernstein and coauthors describe a 1.27 increased odds ratio for NEC in premature infants with IUGR compared to those without growth restriction[5]. Furthermore, infants with IUGR have a different pattern of susceptibility for NEC. While normally grown premature infants are at highest risk for NEC between the postmenstrual ages of 28–32 weeks[7], infants with IUGR remain susceptible past 32 weeks[8]. The mechanisms behind this increased risk remain elusive. In this study, we asked whether IUGR affects the number and/or function of the intestinal epithelial cells performing innate immune cell functions, specifically of goblet and Paneth cells, since they act as a first-line barrier to invading foreign antigens and microorganisms.

To examine the effects of IUGR on the small intestine, we used an infusion of a thromboxane A_2_ (TXA_2_)-analog (U-46619) to elicit maternal hypertension and uteroplacental insufficiency to induce IUGR in a mouse model[9]. Maternal hypertension is the most common cause of human IUGR in developed countries and the most prevalent complication of human pregnancies [10]. We previously demonstrated that IUGR is associated with a decrease in brain, lung, liver, and kidney weights in this mouse model but had not yet examined the effects of IUGR on the developing intestine[9]. We hypothesized that IUGR alters the normal trajectory of intestinal development in this mouse model by affecting intestinal morphogenesis and altering goblet cells and Paneth cells. To ascertain the relevance of our findings, we also investigated goblet and Paneth cell numbers in human IUGR intestinal samples.

## Materials and Methods

### Mice

All animal experiments were performed according to protocols approved by the Institutional Animal Care and Usage Committees at The University of Iowa (Iowa City, IA) and The University of Utah (Salt Lake City, UT). C57BL/6J mice were purchased from The Jackson Laboratory and housed in university-approved vivariums.

### IUGR Mouse Model

**Fig 1:**
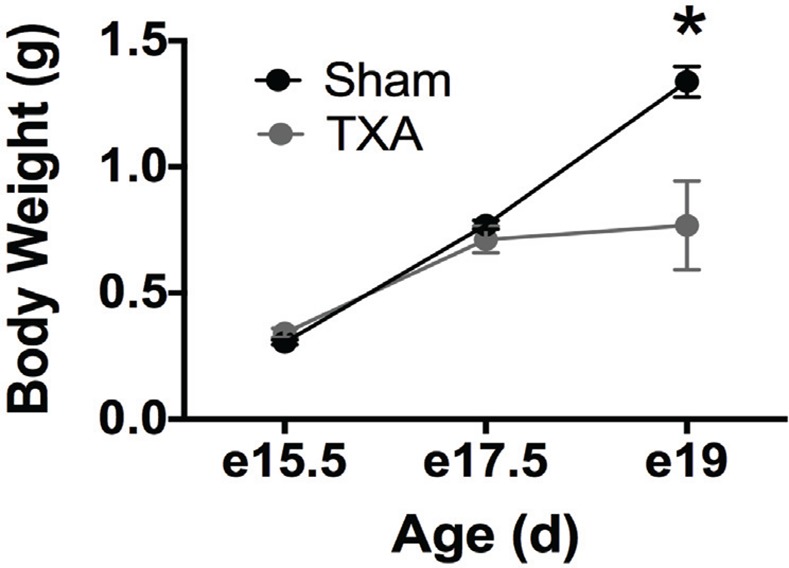
Maternal exposure to U-46619 decreases fetal body weight at E19 compared to sham pups. Pregnant mice were exposed to a TXA_2_- analog (U-46619) or vehicle via micro-osmotic pump infusion from E12.5 until birth (term ~20 days). Body weights of exposed fetuses remained similar to shams at E15.5 and E17.5, but were statistically decreased by E19 (p < 0.001: e15.5 Sham n = 13, e15.5 TXA n = 21, e17.5 Sham n = 8, e17.5 TXA n = 11, e19 Sham n = 5, e19 TXA n = 5).

IUGR was induced in C57BL/6J mice as previously described[9]. In brief, micro-osmotic pumps containing either vehicle (0.5% ethanol) or U-46619 (TXA_2_-analog dissolved in 0.5% ethanol, Cayman Chemical, Ann Arbor, MI) were implanted into anesthetized pregnant dams at embryonic day 12.5 to term (~20 days). U-46619-exposed mouse dams developed hypertension with a mean blood pressure that was 20% higher than sham mouse dams within 24 hours of pump implantation and continued throughout pregnancy. This model produces symmetrically-growth restricted pups that weigh 15% less in birth weight. IUGR pups have similar levels of dehydro-TXB_2_ (a TXA_2_ metabolite), TNF (induced by TXA_2_), and corticosterone as sham pups indicating that IUGR likely originated from hypertension and placental vasoconstriction rather than direct exposure of the drug itself or stress. Using this methodology, embryos from U-46619-exposed dams exhibit significantly lower body weights at e19 compared to sham embryos (Fig 1). As TNF- **β** has been reclassified as lymphotoxin, in this manuscript, TNF refers to the molecule previously known as TNF**-α**.

Following exposure, pups were allowed to deliver spontaneously at term. Unmanipulated dams, set up at the same time as experimental dams, were used to cross foster all sham and IUGR pups until weaning [postnatal day (P) 21] to avoid confounding variables from surgery. Soon after delivery, all pups were weighed. Sham pups that weighed >1.266 g [previously established as above the 10% of weight for the sham population[9]] were kept for cross fostering and constituted the sham group. IUGR pups that weighed <1.266 g were kept for cross-fostering and constituted the IUGR group. Approximately one-third of the offspring born to U-46619 infused dams met criteria for the IUGR group for these studies. To maximize the IUGR phenotype, each age was gated to include offspring of U-46619 infused dams that were below the 10^th^ percentile for weight (small for gestational age) and sham pups above the 10^th^ percentile for weight (appropriate for gestational age).

### Intestinal Harvest and Sample Collection

Intestinal tissue was harvested at P0, P14, P28, and P56 for both sham and IUGR offspring. Fecal contents were removed via manual manipulation followed by flushing with 1 ml PBS. Total body weight, ileal weight, and ileal length were recorded for each animal at the time of harvest. Ileal length was defined as 1/3 the distance from the pyloric junction to the cecum. Each ileum was then divided equally into three sections. The first section was immersed in RNA Later (Life Technologies, Grand Island, NY), stored at 4°C overnight, and then removed and frozen at -80°C. The second section was snap frozen immediately at harvest. The third section was fixed overnight with 10% formalin or Carnoy solution prior to transfer to 70% ethanol at room temperature.

### Human Samples

The Institutional Review Board of Vanderbilt University (Nashville, TN) approved all protocols and samples dealing with human tissue used in these studies. Specific details according to BRISQ recommendations can be found in Table 1[11].

**Table 1:** Human Sample Collection According to BRISQ Criteria.

**Pre-acquisition:**				
Biospecimen type:	Solid tissue			
Collection site:	Tissue taken from the leading edge adjacent to the diseased area of the surgical specimen			
Disease status:	Specimens were obtained from infants who had a surgical disease: atresia, gastroschisis, small intestinal obstruction, or spontaneous ileal perforation. These were then grouped by secondary diagnosis of IUGR or normal growth			
Clinical Data:	Pertinent clinical data obtained were gestational age, age, sex, birth weight, indication for surgical removal and fetal diagnosis (IUGR or normal growth).			
Vital State:	All samples were collected from live patients.			
Diagnosis:	All diagnoses were based on defined surgical or pathological distinctions.			
Time:	All diagnoses required immediate surgical intervention (typically within 24 hours). The exact time between diagnosis and sampling is unknown due to de-identification of samples			
Exposures:	All infants were hospitalized in a single NICU with similar general exposures. Individual differences were not known due to de-identification.			
Reproductive status:	All patients were pre-pubescent.			
Demographics:	Gestational age, age and sex were determined. All other demographics were not available due to de-identification.			
Accrual:	All specimens were obtained as part of Dr. Weitkamp’s bio specimen resource at Vanderbilt that collects surgical tissue samples from all hospitalized infants in the NICU that need an intestinal resection.			
Biobank Info:	Dr. Jörn-Hendrik Weitkamp (hendrik.weitkamp@Vanderbilt.Edu) for more details.			
**Acquisition:**				
Mechanism:	Tissue obtained at the time of surgery was collected in sterile containers and kept in the operating room until completion of the surgical procedure. Subsequently tissue specimens were transported to the surgical pathology laboratory and kept refrigerated until processing (typically within the hour). Surgical pathology staff members removed approximately 1–3 cm from the leading edge of the surgical resection for research purposes.			
Preservation:	This tissue was collected in refrigerated Ca^2+^ and Mg^2+^- free HBSS media with antibiotics (Penicillin, Streptomycin and Gentamicin) and stored in 4^°^C until pick-up by a Weitkamp lab staff member. Upon arrival in the laboratory (within 48 hours), 0.3–1 cm long tissue was removed for long-term preservation and placed in 10% formalin for 24 hours. After that time, tissue was preserved in ethanol and transported to the Vanderbilt tissue processing core for paraffin-embedding. Formalin-fixed and paraffin-embedded (FFPE) tissues were stored in room temperature for max. 1–2 years.			
Storage:	FFPE tissue blocks were stored in room temperature inside a drawer within the Weitkamp laboratory.			
**Quality Assurance:**				
Assessment:	Samples with identifiable villi on microscopic examination and fulfilling study criteria were selected for cutting slides.			
Proximity:	Samples were obtained approximately 1–3 cm from the leading edge of the surgical site			
Enrichment:	Mucosa remained attached, whole tissue sections were collected.			
Embedding:	Paraffin embedding occurred in the Vanderbilt Translational Pathology Shared Resource (TPSR) as follows:			
	70% Alcohol	15 minutes		
	80% Alcohol	15 minutes		
	95% Alcohol	15 minutes		
	95% Alcohol	15 minutes		
	100% Alcohol	15 minutes		
	100% Alcohol	15 minutes		
	Xylene	15 minutes		
	Xylene	20 minutes		
	Wax (paraffin)	30 minutes		
	Wax (paraffin)	20 minutes		
	Wax (paraffin)	15 minutes		
Quality Assurance:	Tissue viability was determined by microscopic examination of H&E stained tissue sections cut from stored tissue blocks.			

In brief, fresh ileum tissue specimens from infants with IUGR or non-IUGR diagnoses were provided from the Vanderbilt Children’s Hospital pathologist under a protocol approved by the Vanderbilt University Institutional Review Board. Written or oral informed consent was waived because all samples were de-identified, not used for diagnostic purposes and therefore would have been otherwise discarded and only demographic data pertinent to the study design (diagnosis and indication for tissue resection, age at time of tissue resection, gestational age, and sex) were collected from patient records prior to tissue release. All human ileal sections were obtained from infants who underwent surgery for non-inflammatory diseases[12, 13]. Control specimens used for our study were taken from preterm infants admitted to the Monroe Carell Jr. Children’s Hospital with non-inflammatory surgical illnesses such as atresia, gastroschisis, small intestinal obstruction, or spontaneous intestinal perforation (n = 9, median gestational age: 36.2 weeks; median age at tissue collection: 8 days). These were compared to infants admitted to the Monroe Carell Jr. Children’s Hospital with a diagnosis of IUGR and a diagnosis of gastroschisis, small intestinal obstruction, or spontaneous intestinal perforation (n = 4, median gestational age for all IUGR infants: 33 weeks; median age at tissue collection: 4 days). The median ages from IUGR and control patients at the time of tissue collection were not statistically different (p = 0.07). All samples were obtained from leading edges of the surgical specimens. Sections were prepared and stained for immunohistochemistry as described below. Additional information regarding either maternal or infant history was unavailable for this study due to de-identification of the human surgical specimens.

### Histology Staining and Immunohistochemistry

Ileal sections were deparaffinized and stained with Alcian Blue/Periodic Acid Schiff (AB-PAS) stain to denote both goblet and Paneth cells as previously described[14, 15]. While Alcian Blue and AB-PAS stains are not specific to goblet and Paneth cells, our experience is that if quantified by a blinded experienced investigator, these stains are equivalent to using cell targeted antibodies but with significantly less background. Goblet cells were quantified per 100 epithelial cells, with greater than or equal to 600 epithelial cells counted per mouse intestine or 300 epithelial cells per human sample. Paneth cells were quantified per crypt, with greater than or equal to 100 crypts counted per mouse intestine or 20 crypts per human intestinal sample. To enumerate the number of proliferating cells, tissue sections were stained with anti-Ki67 antibody (Abcam, Cambridge, MA), and positive cells were quantified per crypt (100 crypts per mouse intestine). Apoptotic cells were determined by tissue staining with rabbit anti-cleaved caspase 3 (R&D MAB835, Minneapolis, MN), as previously reported[16]. A blinded investigator performed all counts using a Nikon NiU microscope using Nikon Elements software (Nikon Inc., Melville, NY). Tight junctions were assessed with anti-zona occludens 1 antibody (Invitrogen, Grand Island, NY). A blinded investigator evaluated all samples, and negative controls were utilized to assess autofluorescence.

### Quantification of mRNA

Ileal samples were homogenized with a TissueLyser LT (Qiagen, Alameda, CA), and RNA was isolated using an RNeasy Plus Mini Kit (Qiagen) according to manufacturer’s directions. RNA concentration was determined using a NanoDrop 1000 Spectrophotometer (Thermo Fisher Scientific, Pittsburg, PA). First-strand cDNA was synthesized using High-Capacity cDNA Reverse Transcription Kit with RNase Inhibitor **(**Life Technologies). Quantitative real-time reverse transcription-polymerase chain reaction (qPCR) was performed using Taqman Fast Universal PCR Master Mix (2X) **(**Life Technologies) and Taqman Gene Expression Assays for muc2 (with assay ID Mm01276696_m1), lysozyme 1 (Mm00657323_m1), cryptdin (Mm025524428_g1), Angiotensin-4 (Mm03647554_g1), Reg3**γ** (Mm00441127_m1), TNF (Mm00443258_m1), IL-6 (Mm00446190_m1), IL-8 (Mm04208136_m1), IL-1**β (**Mm00434228_m1), Occludin (Mm00500912_m1), ZO-1 (Mm00493699_m1), IFN-**γ** (Mm01168134_m1), and Wnt3 (Mm00437336_m1) **(**Life Technologies). Reactions were run in triplicate in a C1000 Thermal Cycler using the CFX96 Real-Time PCR Detection System (Bio-Rad Laboratories, Hercules, CA). 10ng of cDNA were used per reaction. The threshold cycle (Ct) value for each well was obtained by using the instrument’s software. Fold change in gene expression was determined by normalizing gene expression to β-actin in each sample. The 2**ΔΔ–**CT method was used to compare gene expression levels between samples.

### Statistical Analysis

Statistical analysis was performed with PRISM 6.0 software (GraphPad Software, La Jolla, CA). Statistical differences were declared at p<0.05 and were determined using ANOVA or linear regression as appropriate. Differences between multiple comparisons were determined using Holm-Sidak or student t-test where appropriate. All experiments were performed in triplicate.

## Results

### Weights and Lengths

**Fig 2:**
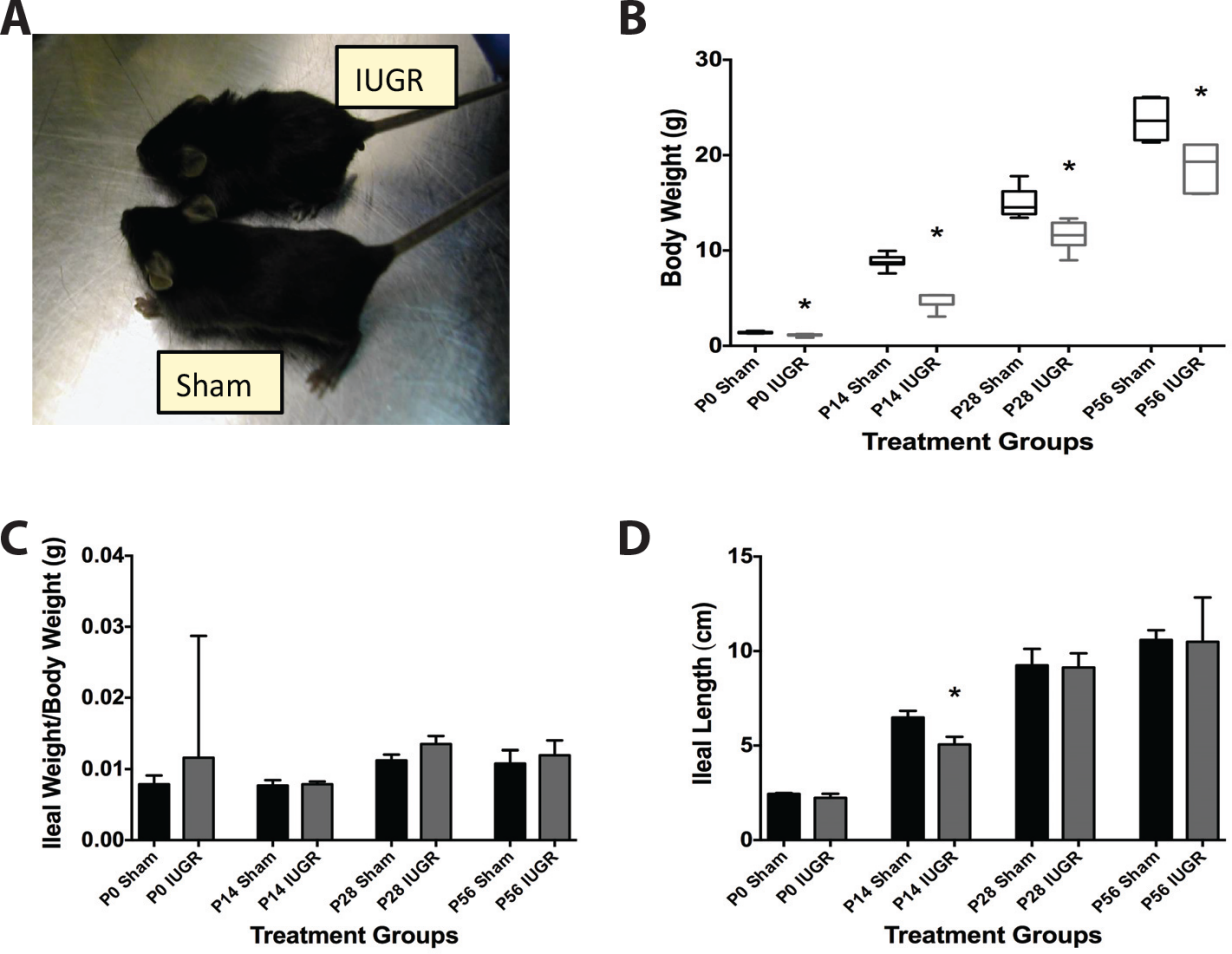
IUGR pups have decreased body weight and ileal length compared to shams. Pregnant mice were exposed to a TXA_2_-analog or vehicle via micro-osmotic pump infusion, and pups were delivered and raised by cross-foster dams. (A) Representative picture of a weaned P21 sham and IUGR mouse to illustrate size differences. (B) Body weights of IUGR pups were significantly less than sham pups from birth to P56 (p < 0.001: P0 Sham n = 15, P0 IUGR n = 12, P14 Sham n = 14, P14 IUGR n = 6, P28 Sham n = 12, P28 IUGR n = 8, P56 Sham n = 7, P56 IUGR n = 7). (C) Harvested ileal weights were similar in both sham and IUGR groups, but (D) ileal lengths were significantly shorter in P14 IUGR pups compared to sham pups (p = 0001, n for each treatment group was the same as Fig 2B).

As described above in **Materials and Methods,** pups from U-46619 treated dams that weighed <1.266 g at birth were defined as IUGR animals. These pups had birth weights of 1.10 ± 0.10 g, versus 1.40 ± 0.07g for the sham group. Lower body weight was maintained at P14, P28, and P56 compared to age-matched sham pup weights (n>6 in each group, Fig 2A and 2B.) When normalized to body weight, ileal weights were not different between sham and IUGR pups at any time point (Fig 2C). However, IUGR mice demonstrated a significant decrease in ileal length at P14 (p = 0.001) compared to sham mice with normalization by P28 (Fig 2D).

### Goblet Cells

**Fig 3:**
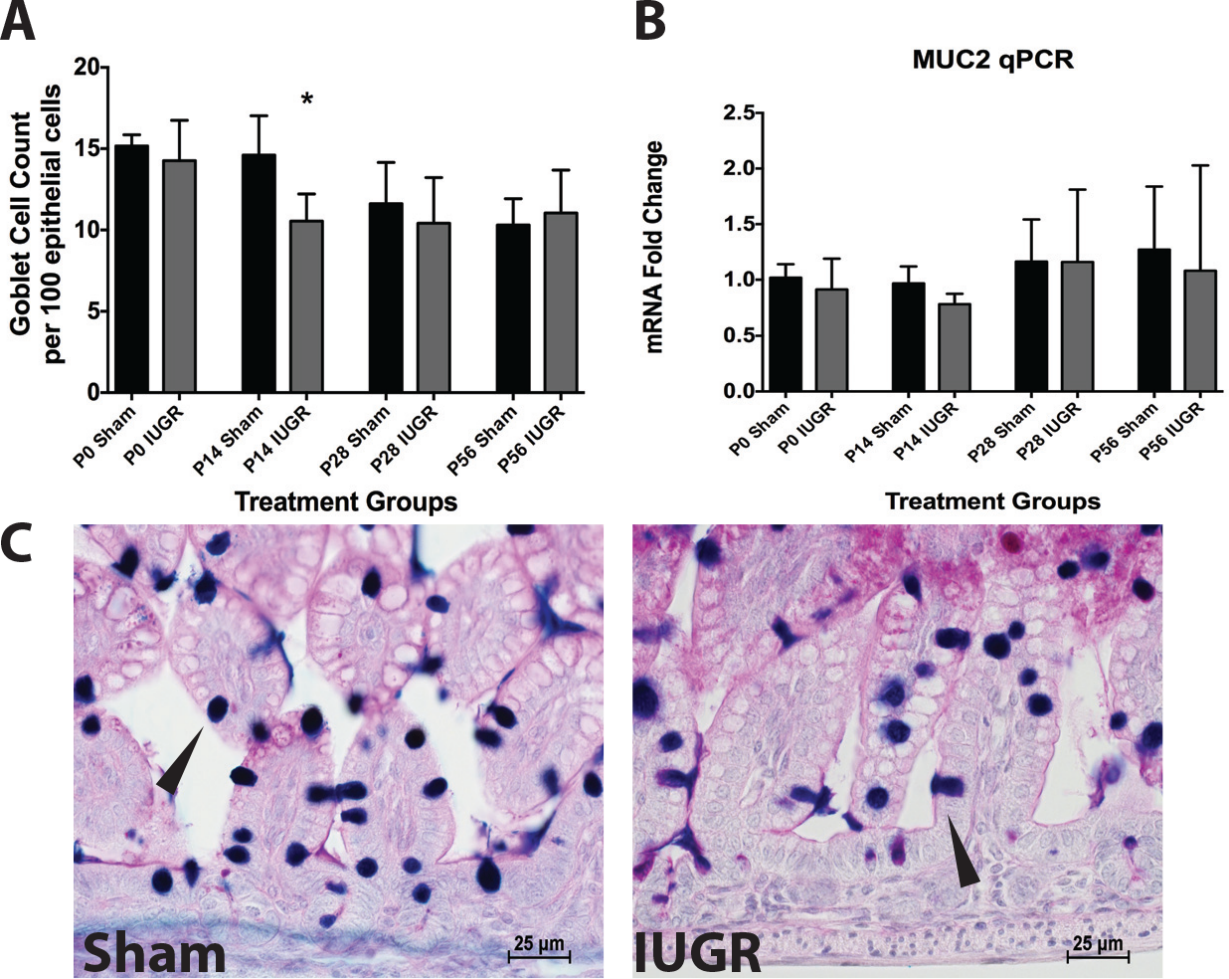
IUGR pups have reduced goblet cell counts at age P14 compared to sham controls. (A) Mucus-positive goblet cells were quantified per 100 epithelial cells at various ages in IUGR and sham mice (P0 Sham n = 13, P0 IUGR n = 11, P14 Sham n = 14, P14 IUGR n = 6, P28 Sham n = 12, P28 IUGR n = 8, P56 Sham n = 5, P56 IUGR n = 7). IUGR mice at P14 had significantly fewer goblet cell/epithelial cell ratios compared to sham mice (p = 0.001). (B) Despite differences in mucus-positive goblet cell numbers at P14, no differences were noted in MUC2 mRNA levels (p = 0.9: P0 Sham n = 4, P0 IUGR n = 4, P14 Sham n = 9, P14 IUGR n = 5, P28 Sham n = 10, P28 IUGR n = 6, P56 Sham n = 7, P56 IUGR n = 7). (C) Representative micrographs from P14 sham and IUGR mice taken with a 40x objective with goblet cells denoted by arrows.

IUGR mice had significantly decreased mucus positive goblet cell counts at P14 compared to sham (p = 0.0011) (Fig 3A). The mRNA levels of MUC2, the major mucin gene expressed by goblet cells, were similar in sham and IUGR pups at all time points (Fig 3B).

### Paneth Cells

**Fig 4:**
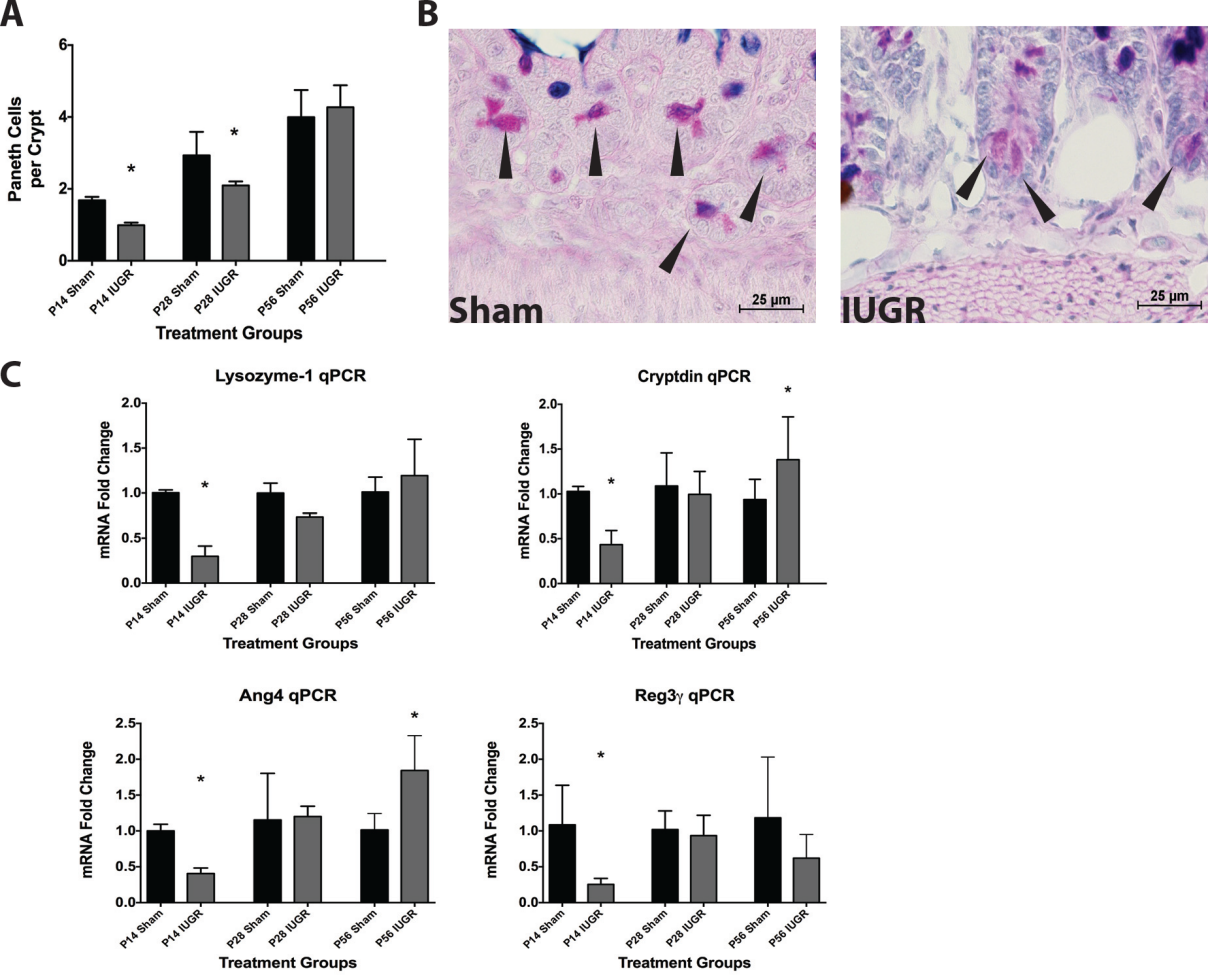
IUGR pups have reduced Paneth cell counts gene products compared to sham controls. (A) Granule-positive Paneth cells were quantified per crypt at different ages in IUGR and sham mice (P14 Sham n = 14, P14 IUGR n = 5, P28 Sham n = 12, P28 IUGR n = 6, P56 Sham n = 7, P56 IUGR n = 7). IUGR mice had significantly fewer Paneth cell counts at P14 (p = 0.01) and P28 (p = 0.002) compared to P14 shams. (B) Representative micrographs of P14 mice taken with a 40x objective showing Paneth cells as denoted by arrows in sham and IUGR. (C) IUGR mice also had significantly reduced lysozyme 1 (p<0.0001), cryptdin (p = 0.003), Ang4 (p = 0.017), and Reg3**γ** (p = 0.024) mRNA levels at P14. In contrast, cryptdin and Ang4 mRNA levels were significantly increased in P56 IUGR mice compared to sham mice (p = 0.02 and 0.003 respectively: P14 Sham n = 9, P14 IUGR n = 5, P28 Sham n = 7, P28 IUGR n = 6, P56 Sham n = 7, P56 IUGR n = 7).

We examined intestines for this cell type from P14 onward as they are not present prior to this age in mice[17, 18]. IUGR mice had significantly fewer Paneth cells per crypt at P14 and P28 (p = 0.0136 and 0.0023, respectively) compared with age-matched sham mice (Fig 4A). This decrease disappeared by P56. In association with cell number decrease, IUGR significantly decreased mRNA levels of lysozyme 1, cryptdin, angiotensin 4 (Ang4), and regenerating islet-derived protein 3 gamma (Reg3**γ**) (Fig 4C), which are all markers of Paneth cells, at P14 compared to sham. These decreases were resolved by P28. At P56, IUGR ileum showed a significant increase in cryptdin and Ang4 mRNA compared to sham (Fig 4C).

### Cell Proliferation and Apoptosis

**Fig 5:**
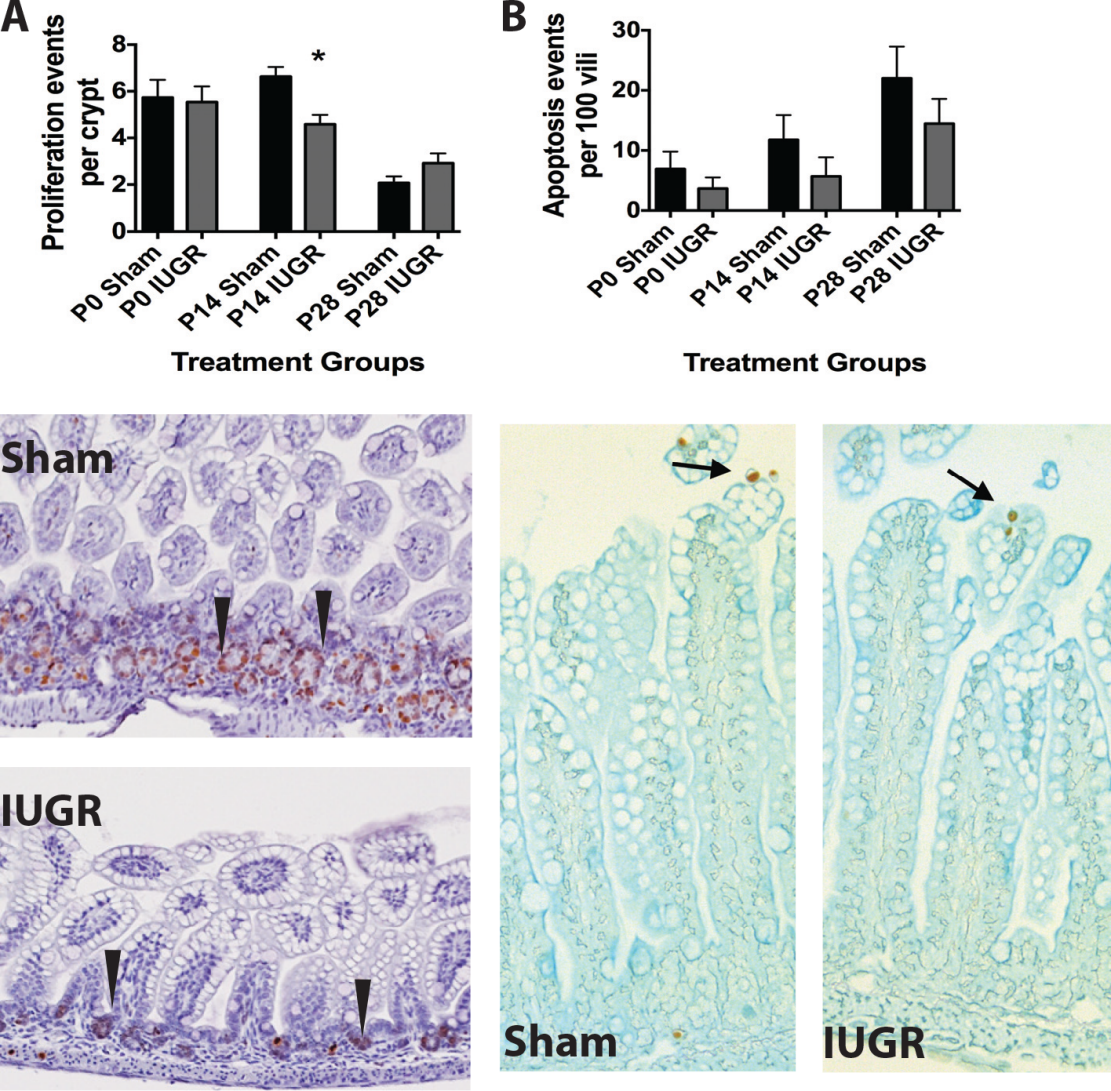
IUGR pups have decreased cell proliferation at age P14 compared to sham controls. (A) Actively proliferating cells were stained with anti-Ki67 antibody and the number of Ki-67 positive cells per intestinal crypt was quantified in IUGR and sham mice. IUGR decreased the number of Ki-67^+^ cells at P14 only compared to shams (n = 5 for each treatment group, p = 0.04, representative micrographs from P14 mice are shown as the lower panel of A). (B) Apoptotic cells were stained with anti-cleaved caspase-3 antibody and the number was quantified in IUGR and sham mice. IUGR did not affect the number of apoptotic events per 100 vili at any time point (n = 3 for each treatment, p = 0.6, representative micrographs are shown from P28 mice as the lower panel of B).

To examine the homeostatic processes of proliferation and apoptosis in the ileum, we quantified Ki67 and cleaved caspase-3 positive cells respectively. IUGR mice had fewer proliferating cells per intestinal crypt at P14 compared to sham (Fig 5A). No significant change in apoptosis was observed at any age after IUGR (Fig 5B).

### Generalized inflammation and barrier function

**Fig 6:**
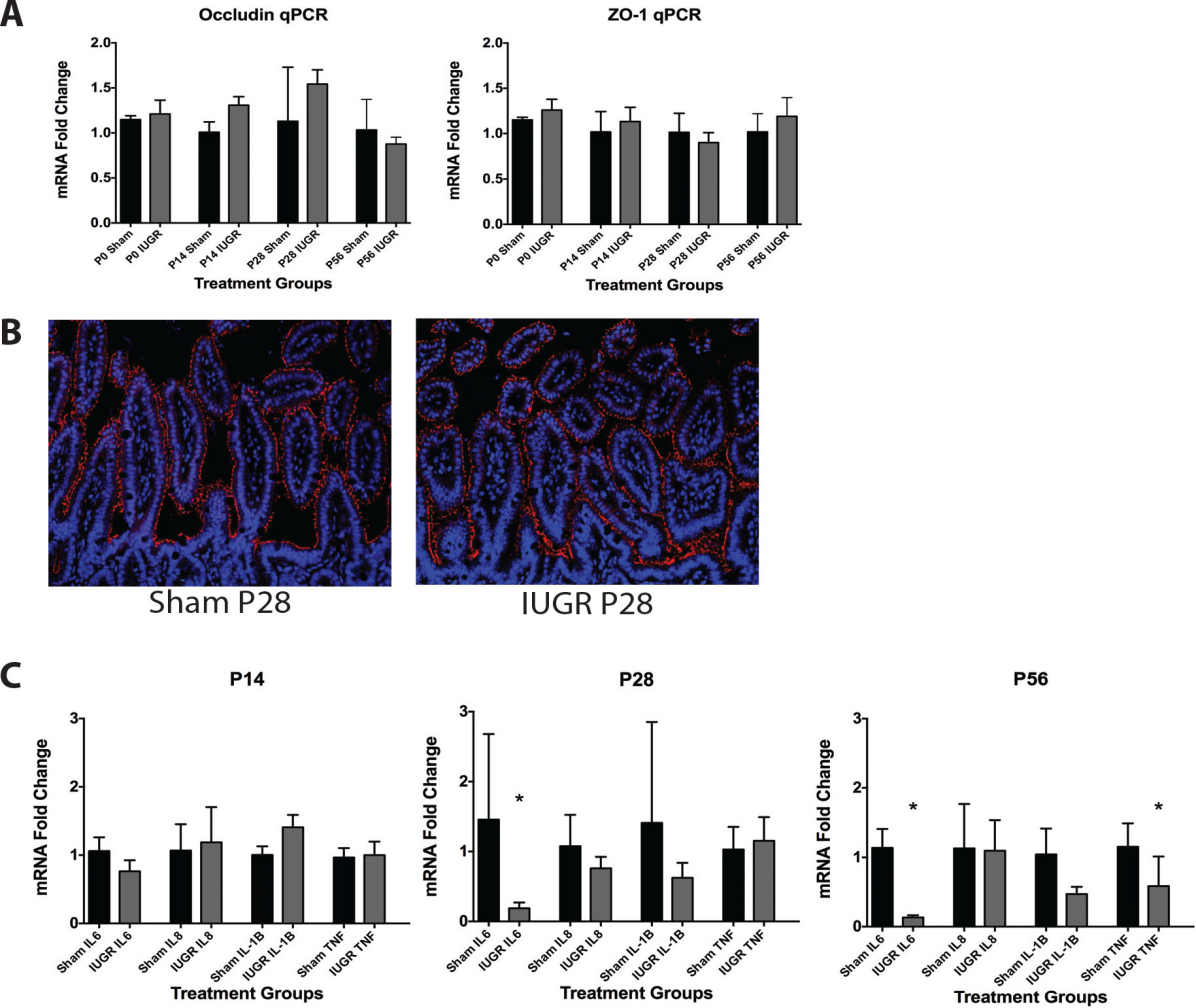
IUGR pups show altered mRNA levels compared to sham controls. (A) Expression of mRNA was measured using qRT-PCR. TXA_2_ analog-induced IUGR caused no significant change in mRNA levels of either occludin or ZO-1 at any age (Fig 6A, n = 4 for all treatment groups). (B) Immunohistochemistry of ileal samples showed no differences in quantity or localization of the tight junction protein ZO-1. ZO-1 staining is shown in red, and nuclei as marked by DAPI staining are shown in blue (Fig 6B, n = 3 for all treatment groups, samples from P28 sham and IUGR animals are shown). (C) TXA_2_ analog-induced IUGR had no effect on mRNA levels of IL-6, IL-8, IL-1**β**, or TNF at P14, but caused significant decreases in IL-6 at P28 and P56, and in TNF at P56 (p = 0.02, 0.004, and 0.02 respectively, n = 4 for all treatment groups).

To determine if TXA_2_ analog-induced IUGR would affect the intestine barrier integrity and inflammatory function of the developing mouse pups, we measured gene expression of two key intestinal tight junctional proteins, ZO-1 and occludin, as well as several cytokines involved in intestinal inflammation including IL-1**β**, IL-6, IL-8, and TNF. There were no significant changes in mRNA observed in either of the tight junctional proteins (Fig 6A). To examine if the localization of tight junction proteins differed, ileal samples were stained for presence of ZO-1. Again we found no significant differences in presence or localization of ZO-1 (Fig 6B). As for our inflammatory cytokines, while no significant changes were detected in IUGR pups at P14, IL-6 expression was significantly decreased at P28, while both IL-6 and TNF were significantly decreased at P56 (Fig 6C).

### Correlating gene expression levels with IUGR severity

**Fig 7:**
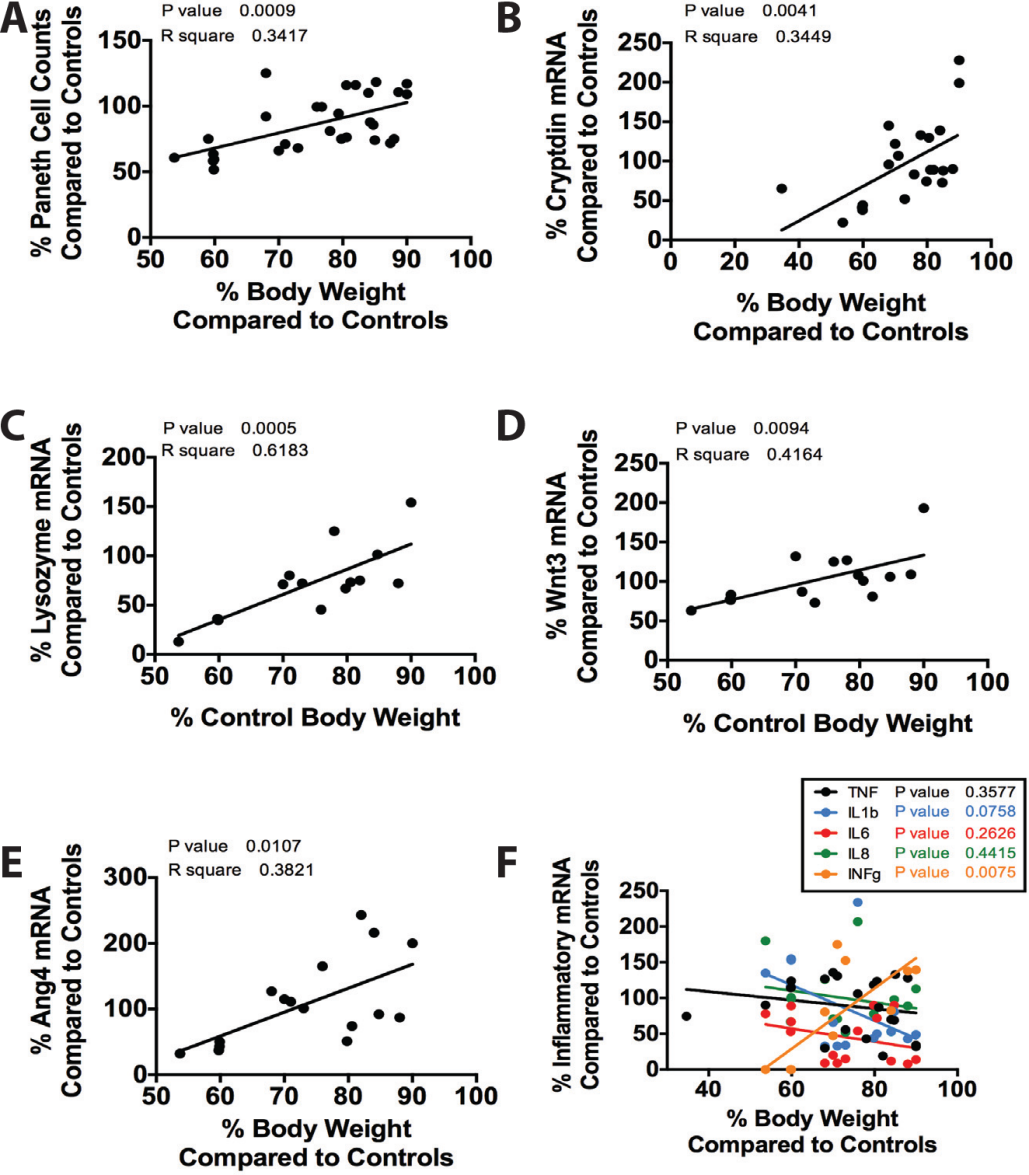
The severity of IUGR significantly impacts its effect on Paneth cells. Data obtained from prior experiments were calculated as a percentage of change from the average control value of the same age. Data were plotted as deviation in weight compared to change in the desired variable and significance was determined using linear regression. There was a significant correlation between severity of IUGR and (A) Paneth cell loss (n = 29, p = 0.0009, r^2^ = .34), (B) decrease in cryptdin mRNA expression (n = 22, p = 0.0041, r^2^ = .34), (C) decrease in lysozyme mRNA (n = 15, p = 0.0005, r^2^ = 0.62), (D) decrease in Wnt3 mRNA (n = 15, p = 0.0094, r^2^ = 0.42), (E) decrease in Ang4 mRNA (n16, p = 0.012, r^2^ = 0.38), (F) and decrease in INF-**γ** mRNA (n = 15, p = 0.0075, r^2^ = 0.56). (F) While statistically non-significant, there were trends towards increased mRNA levels of TNF, IL-1**β**, IL-6, and IL-8, which are all pro-inflammatory cytokines seen in inflammatory intestinal diseases.

Since TXA_2_ analog-induced IUGR had significant effects on several important genes, we wondered if the gene expression levels correlated with the severity of IUGR. To examine this, we divided each IUGR data point by the control average for the same age. Data were then plotted as a function of percentage of change in body weight from control compared to percentage in change from the experimental variable. This experimental design was constructed to examine if severity in IUGR would correlate with changes in outcomes such as cell counts or mRNA expression levels while minimizing the effect of developmental age. Significance was determined using linear regression statistics. We found that the more severe the growth restriction, the more significant the loss of Paneth cells. We also found that the more severe the growth restriction, the larger the decrease in mRNA expression of cryptdin, lysozyme, Wnt3, Ang4, and INF-**γ**. Whilst statistically insignificant, there were additionally trends toward increased mRNA expression of TNF, IL-1**β**, IL-6, and IL-8 with increased severity of IUGR (Fig 7).

### Goblet and Paneth Cells in Humans with IUGR

**Fig 8:**
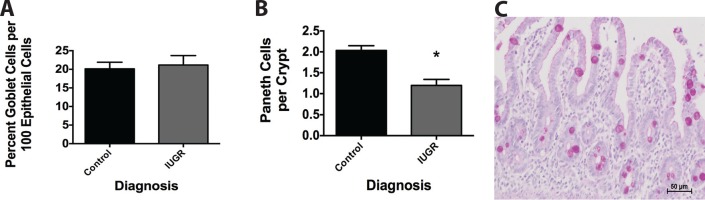
Human preterm infants diagnosed with IUGR have decreased Paneth cell counts compared to preterm non-IUGR control infants. Tissues obtained from preterm infants with non-inflammatory surgical small intestinal diseases were stained for presence of goblet and Paneth cells. Infants with a diagnosis of IUGR (n = 9) were compared to age-matched non-IUGR infants (n = 4). (A) IUGR infants had similar numbers of goblet cells per 100 epithelial cells compared to non-IUGR infants (p = 0.5). (B) IUGR infants had decreased numbers of Paneth cells per crypt compared to non-IUGR infants (p = 0.04). (C) Shown is a representative micrograph of human tissue taken with a 20x objective and stained with Alcian Blue-PAS staining.

To determine if our mouse findings correlated with human IUGR intestines, we quantified mucus-positive goblet cells and granule-positive Paneth cells in tissues obtained from preterm infants with non-inflammatory surgical small intestinal diseases (atresias and obstructions). Preterm infants with IUGR were compared to age matched preterm non-IUGR infants. Human IUGR infants had similar numbers of goblet cells per 100 epithelial cells as control infants (Fig 8A). In contrast, we found fewer Paneth cells per crypt in IUGR infants compared to control infants (Fig 8B).

## Discussion

IUGR infants are at increased risk for short- and long-term health complications affecting multiple organ systems including the gastrointestinal tract. Specifically, IUGR infants have an increased susceptibility to feeding intolerance and NEC[2, 4, 5]. Our laboratory has previously demonstrated that appropriately grown preterm human infants with NEC have decreased goblet and Paneth cells[17, 19], but little is known about the developmental trajectory of goblet and Paneth cells after IUGR. We therefore hypothesized that the increased susceptibility to gastrointestinal disease witnessed following IUGR is in part due to alterations in intestinal maturation and a disruption of the innate immunity in the IUGR intestine. Using a mouse model of IUGR that mimics human pregnancy-induced hypertension, we specifically hypothesized that IUGR decreases the number of mucus-producing goblet and antimicrobial-secreting Paneth cells. We demonstrated that IUGR alters both the growth and the cellular composition of the developing small intestine. Specific findings include shortened ileal length, fewer Paneth cells, a reduction in Paneth cell specific mRNA abundance in mid intestinal development (P14) followed by a generalized increase in the same mRNA abundance toward adulthood (P56). We also demonstrated that the more growth restricted an offspring is, the larger the decrease in Paneth cell counts and secretory products as well as a larger increase in the mRNA expression of inflammatory genes.

The development of the small intestine follows a conserved pattern in all mammals. This pattern begins with an initial morphogenesis of the simple tubular structure followed later by invaginations into villi and crypt structures[20–22]. As the villi and crypts evolve, cellular differentiation occurs along the single layer of epithelial cells[21]. While the intestine is often thought of as a means for nutrient absorption, it must also function in a protective role as it is constantly exposed to foreign antigens and microorganisms. Therefore, the epithelial layer has evolved to contain cells specialized for such roles as nutrition (enterocytes), hormonal regulation (enteroendocrine cells), and critical innate immunity (goblet and Paneth cells)[23].

Goblet cells are the major secretory cell of the intestine. Goblet cells produce mucus, which create a structural barrier to entrap pathogens and prevent them from contacting the epithelium, and helps assist with peristalsis-driven bacterial removal[24]. Goblet cell dysfunction in adults has been linked to inflammatory diseases including colitis and cancer[25]. The major mucin produced by small intestinal goblet cells is Muc2. Deficiencies in Muc2 have been seen in both human[12] and animal models of NEC[26]. Our data shows that TXA_2_ analog-induced IUGR induces a significant decrease in P14 animals compared to controls. While our histologic staining technique of using Alcian Blue is not goblet cell specific, our past experience has found that it is an equivalent marker to using anti-MUC2[12, 14, 15, 17]. This decrease is then compensated for, as IUGR animals at P28 are not significantly different from controls. Interestingly, the expression of MUC2 is not altered at any time point. This discrepancy between mucus present in the goblet cells and mRNA expression is likely due to the stage of development, as immature (as opposed to more adult) small intestine will secrete mucus without a compensatory upregulation of MUC2 mRNA expression[12].

Paneth cells are antimicrobial peptide secreting cells that serve to regulate microbial flora, maintain stem cell homeostasis, and assist with host defense[27]. Paneth cell abnormalities have been linked to susceptibility to inflammation in intestinal diseases such as Crohn’s disease[28]. Our data demonstrate a disruption in Paneth cells in the setting of IUGR during a critical window of intestinal development. Specifically, granule-containing Paneth cells were significantly decreased in TXA_2_ analog-induced IUGR animals at P14 and P28 compared to controls. While our histologic staining technique of using AB-PAS is not Paneth cell specific, our past experience has found that it is an equivalent marker to using anti-lysozyme[12, 14, 15, 29]. This loss of Paneth cells was mirrored by significant decreases in multiple genes found in Paneth cells. A loss in number and/or function of these cells suggests a loss of innate immunity in the immature small intestine, which has also been hypothesized to play a role in development of inflammatory diseases such as NEC[30]. What is more interesting is that, despite no change in Paneth cell numbers in IUGR animals compared to controls at P56, we saw significant increases in both cryptdin and Ang-4 mRNA at P56. Both cryptdins and Ang-4 represent antimicrobial peptides produced by Paneth cells. This suggests a strong effort by the intestine to normalize Paneth cell function, and may also represent a change in the adult phenotype induced by stimuli that occur in the fetal period. Lastly, we wanted to test if there was a correlation between the severity of IUGR and the effects on the intestinal tract development. While our sample sizes were too small to confirm our correlations in humans, we found a strong correlation between the severity of growth restriction and disruption of Paneth cells.

In addition to its effects on goblet and Paneth cells, IUGR alters the normal homeostasis of the intestinal epithelial layer. Our data show a significant decrease in cellular proliferation at P14, correlating with significant decreases in goblet cells and Paneth cells. Since proliferative cells within the epithelium include stem/progenitor cells, our results suggest that in the immature IUGR intestine intestinal stem cells may have decreased regenerative ability. This limited ability to repair damage may further compound intestinal injury when the intestine is faced with insults later during development. While cellular proliferation was altered by induction of IUGR, we saw an expected pattern of minimal apoptosis located at the vilus tips with no differences between sham and IUGR animals at any age. In addition to a potential decreased regenerative ability of stem/progenitor cells, our correlation data (Fig 7D) shows that as severity of IUGR increases (and body weight decreases), there is a significant association with decreased mRNA expression of Wnt3. Wnt3 has been shown to be a key cellular signal for regulation of intestinal stem cell homeostasis and differentiation[31–33]. In particular, the Wnt/**β**-catenin signaling has different effects on the development of the secretory cell types of Paneth cells and goblet cells. Paneth cells require Wnt and leucine-rich repeat-containing receptor 4 (Lgr4) whereas high Wnt activity interferes with goblet cell differentiation[34–36]. Therefore, the more persistent decrease in Paneth cells up to P28 over goblet cells may reflect decreased Wnt3 activity amidst decreased proliferative ability. Further studies in intestinal stem cell reporter mice are needed to verify these data. Lastly, we briefly examined the effect of IUGR on tight junctional proteins in the small intestinal epithelium. IUGR had no effects on gene expression of ZO-1 or occludin or the localization of ZO-1. While we found no differences in our experiments, the epithelial junctional complexes are a dynamic system of many proteins and can be greatly influenced by environmental factors such as inflammation. Thus, future experiments involving a second “inflammatory” insult such as with our laboratory’s model of necrotizing entercolitis[15, 17] in addition to IUGR will help to further establish the effects of IUGR on the epithelial barrier system.

Additional factors likely affecting IUGR-induced intestinal pathogenesis warrant future study. Nutrient sufficiency is an important variable to ensure normal intestinal development. We attempted to control for postnatal nutritional intake by cross-fostering all sham and IUGR offspring within 24 hours of delivery to unmanipulated dams generated at the same time as dams designated for sham and IUGR surgeries. Even though significant intestinal maturation occurs prenatally in humans but postnatally in mice, both human and mouse development occur with a similar progression of developmental events [37], therefore data gleaned from murine experiments can help to inform the human experience. Our archived human samples were also de-identified and limited in number, therefore additional information cannot be gathered at this point. Despite these challenges, there were key similarities between the mouse model and human IUGR, particularly in the delayed development of mature Paneth cells, which may provide a window of vulnerability in which disease could take root.

Another important factor impacting the immunity of the IUGR infant is the host-microbiome interaction. Clearly, the role of the intestinal microbiome has a significant impact on both homeostasis and pathology[29]. While attempts to look at the effects of IUGR on the microbiome were outside the scope of this work, they must be considered when thinking about the host immune system. This consideration is especially relevant in light of our findings that inflammatory genes such as IL-6 and TNF were significantly down-regulated in TXA_2_ analog-induced IUGR.

Our findings show shorter villus length, decreased numbers of Paneth and goblet cells, decreased proliferation, and decreased gene expression; this suggests one of two potential mechanisms. First, TXA_2_ analog-induced IUGR animals may be showing a protective instead of homeostatic phenotype[14]. Instead of spending energy and cellular recourses to generate new intestinal cells and provide nutrition to the host, the epithelial layer is more dormant, awaiting the passing of a danger signal. A second possibility is that TXA_2_ analog-induced IUGR animals retain a more immature phenotype for longer than their normally grown counterparts. For example, the villus length of P14 IUGR animals is remarkably similar to normally grown P7 animals. This suggests that Paneth cells are not simply lost, but their development lags behind in the IUGR phenotype. Development of cell surface specific antibodies in the future will help clarify this issue.

In conclusion, we demonstrate that IUGR decreases the innate immune cell composition and function in the developing mouse small intestine. These findings correlate with human IUGR intestinal findings. We have also demonstrated that IUGR mice have decreased proliferative capacity at a time when mature cell types are also diminished. Together, these changes in the mouse innate intestinal immunity suggest a possible mechanism behind the increased susceptibility of infants with intrauterine growth restriction to develop intestinal diseases such as NEC.
